# Rapamycin Promotes Mouse 4T1 Tumor Metastasis that Can Be Reversed by a Dendritic Cell-Based Vaccine

**DOI:** 10.1371/journal.pone.0138335

**Published:** 2015-10-01

**Authors:** Tien-Jen Lin, Wen-Miin Liang, Pei-Wen Hsiao, Pradeep M. S, Wen-Chi Wei, Hsin-Ting Lin, Shu-Yi Yin, Ning-Sun Yang

**Affiliations:** 1 Graduate Institute of Injury Prevention and Control, Taipei Medical University, Taipei, Taiwan, ROC; 2 Department of Neurosurgery, Taipei Medical University—Wan Fang Hospital, Taipei, Taiwan, ROC; 3 Agricultural Biotechnology Research Center, Academia Sinica, Taipei, Taiwan, ROC; 4 Graduate Institute of Biotechnology, National Chung Hsing University, Taichung, Taiwan, ROC; 5 Biostatistics Center, China Medical University, Taichung, Taiwan, ROC; 6 Institute of Environmental Health, Department of Public Health, School of Public Health, China Medical University, Taichung, Taiwan, ROC; 7 Taiwan International Graduate Program (TIGP), Molecular and Biological Agricultural Sciences Program, Academia Sinica, Taipei, Taiwan, ROC; University of Tennessee Health Science Center, UNITED STATES

## Abstract

Suppression of tumor metastasis is a key strategy for successful cancer interventions. Previous studies indicated that rapamycin (sirolimus) may promote tumor regression activity or enhance immune response against tumor targets. However, rapamycin also exhibits immunosuppressant effects and is hence used clinically as an organ transplantation drug. We hypothesized that the immunosuppressive activities of rapamycin might also negatively mediate host immunity, resulting in promotion of tumor metastasis. In this study, the effects of rapamycin and phytochemical shikonin were investigated *in vitro* and *in vivo* in a 4T1 mouse mammary tumor model through quantitative assessment of immunogenic cell death (ICD), autophagy, tumor growth and metastasis. Tumor-bearing mice were immunized with test vaccines to monitor their effect on tumor metastasis. We found that intraperitoneal (ip) administration of rapamycin after a tumor-resection surgery drastically increased the metastatic activity of 4T1 tumors. Possible correlation of this finding to human cancers was suggested by epidemiological analysis of data from Taiwan’s National Health Insurance Research Database (NHIRD). Since our previous studies showed that modified tumor cell lysate (TCL)-pulsed, dendritic cell (DC)-based cancer vaccines can effectively suppress metastasis in mouse tumor models, we assessed whether such vaccines may help offset this rapamycin-promoted metastasis. We observed that shikonin efficiently induced ICD of 4T1 cells in culture, and DC vaccines pulsed with shikonin-treated TCL (SK-TCL-DC) significantly suppressed rapamycin-enhanced metastasis and Treg cell expansion in test mice. In conclusion, rapamycin treatment in mice (and perhaps in humans) promotes metastasis and the effect may be offset by treatment with a DC-based cancer vaccine.

## Introduction

Rapamycin has been extensively studied in recent years and is known to exhibit multiple biochemical and medicinal activities including anti-bacterial, anti-fungal and immunosuppressive effects, rapamycin can also inhibit antibody formation and antigen-induced B cell and T cell proliferation activities.[1] Because of these characteristics, rapamycin has been developed into a commercially used immunosuppressant, prophylaxis drug for use in patients following organ transplantation,[1] and is approved by the US Food and Drug Administration (FDA) for renal rejection.

When tested against the National Cancer Institute (NCI) 60 tumor cell line panel, rapamycin inhibited the growth of a number of tumor cell lines including colon, mammary and skin carcinoma cells.[2] This drug is well known for conferring specific anti-mTOR activity under various in vivo and in vitro conditions.[2] Recognition of rapamycin as a target therapy for blocking the mTOR pathway has also led to the development of rapamycin analogues as potential chemotherapeutic agents against solid tumors, including breast cancers.[3] The mammalian target of rapamycin complex 1 (mTORC1) is a well-recognized master regulator of cell growth and proliferation.[4, 5] Some recent studies have suggested that constitutive activation of mTORC1 in normal cells could lead to development of malignant tumors in a variety of tissues, and rapamycin can arrest cell cycling at the G1 phase via binding to the mTORC1 target.[6] It is also reported to inhibit metastasis of human renal cancers.[7] Rapamycin in combination with letrozole was evaluated in a phase III clinical trial for metastatic breast cancers. This combination, however, was not shown to be more beneficial than letrozole alone.[8] Due to the various potential applications of rapamycin for anti-tumor activities, possible side effects such as promotion of tumor metastasis are serious concerns, but to the best of our knowledge, have not been thoroughly investigated to date.

Immunogenic cell death (ICD) of tumor cells and the derived tumor cell lysates (TCL) have been shown to induce effective anti-tumor immune responses through activation of dendritic cells (DCs) and the consequent activation of specific T cell responses.[9] The FDA has approved such a DC-based therapeutic vaccine for the treatment of specific prostate cancers.[10] We reported recently that phytochemical shikonin can effectively induce ICD and enhance the immunogenicity of TCL (termed SK-TCL) derived from treated mouse tumor cells.[11] The combined SK-TCL and LPS treatment can activate DCs to high maturation status and enhance the priming of Th1/Th17 effector cells. When this SK-TCL formulated DC vaccine was used to treat test mice, CD86 and MHC class II were expressed at high levels, effectively activating Th1/Th17 cells and resulting in strong anti-metastatic activities.[11]

mTORC1 plays a key role in the regulation of autophagy by phosphorylating components of the autophagy-induction machinery.[12] Immunization of mice with purified tumor-derived autophagosomes was shown to induce tumor-specific T-cell responses.[13] For tumor cell invasion, regulatory T cells (Tregs) are known to play a key role in the metastatic escape of cancer cells from the antitumor effector T cells.[14] Rapamycin can enhance the expansion of CD4 Foxp3 Tregs and this can result in suppression of other CD4 T cell activities.[15]

In this study, first, we report that rapamycin activates the expansion or/and differentiation of Treg cells, which may promote the metastasis of test 4T1 mammary carcinoma cells in test mice. Such promotion of metastasis by rapamycin can be facilitated by activation of Th1/Th17 associated activities. We next demonstrate that our previously developed shikonin-tumor cell lysate (SK-TCL)-loaded DC-based cancer vaccines can help alleviate or offset such rapamycin-promoted 4T1 tumor metastasis. Consistently, a human epidemiology database analysis of cancer patients in Taiwan also revealed a positive correlation between prior clinical use of rapamycin and the cancer incidence rate. Our findings from this study raised questions and measures about future clinical application and implication of rapamycin usage.

## Materials and Methods

### Compounds

Shikonin (SK) was purchased from Tokyo Chemical Industry (Tokyo, Japan). Rapamycin was from LC Laboratories (St. New Boston, MA, USA), and doxorubicin (DOX) was from Sigma (St. Louis, MO, USA).

### Cell lines and preparation of tumor cell lysates

4T1 mammary tumor cells were obtained from Dr. Pei-Wen Hsiao (ABRC, Academia Sinica, Taipei) and were originally derived from ATCC (Nov. 11, 2003, lot No. 3306022 CRL-2539). The 4T1 cells from ATCC were examined with Molecular Testing of Biological Materials (MTBM) test (Animal Health Diagnostic Laboratory, NCI-Frederick) on Nov. 25, 2003 and Oct.27, 2010, and 4T1 ells from Dr. Hsiao was lastly tested for their morphology, in vitro and in vivo growth rate and their metastatic ability on Feb. 24, 2014.

Mouse mammary carcinoma 4T1-luc2 (i.e., 4T1 cells transfected by a firefly luciferase cDNA gene) cell lines [16] also were kindly provided by Dr. Hsiao, and these cells were employed in the spontaneous metastasis experimental model after a surgical resection of the primary tumor. The latest evaluation for bioluminescence signals from implanted 4T1-luc2 tumor cells in test mice was performed on April 6, 2014. As analyzed using a non-invasive IVIS imaging system (Calipers, Hopkinton, MA). Both 4T1 and 4T1-luc2 cells were maintained in RPMI-1640 complete medium (i.e., RPMI-1640 supplemented with 10% FBS, 100 μM non-essential amino acids, 100 μM sodium pyruvate, 100 μg/ml streptomycin and 100 unit/ml penicillin) and grown in a 5% CO_2_ incubator at 37°C. Various 4T1 TCL samples derived from phytochemical-treated tumor cells were prepared as described previously.[16] Briefly, after cells were grown to 50% confluence and treated with test compounds, 4T1 cells were collected and resuspended in PBS at 24 h or 48 h post-treatment with different test compounds, for induction of ICD. Test cells in suspension were first frozen in liquid nitrogen for 1.5 min, then thawed and sonicated for 4 min at 4°C. The freeze–thaw cycles were repeated four more times. After the final thaw, TCL suspensions were sonicated to further disrupt the aggregates/debris in the cell suspension. Prior to use, cell lysates were thawed and centrifuged at 12,000 rpm for 30 min, and the supernatant was used as the source of tumor antigen. Lysates were frozen at -80°C until use.

### Collection of rapamycin- and shikonin-induced autophagosome organelle fractions

Aliquots of 4T1 cells were treated with 5 μM shikonin or rapamycin for 48 h in culture. Autophagosome (AS)-enriched fractions were isolated as described previously.[17] Conditioned culture media were collected and cleared of dead cells and cell debris by centrifugation (300 × g, 10 min). To obtain the autophagosome-enriched organelle fractions (Rapa-AS and SK-AS), medium supernatants containing the crude large vesicles were centrifuged at 10,000 × g for 15 min. The partially purified AS pellet was resuspended in PBS and the protein concentration determined. For the control [vehicle (PBS)-treated] cell treatment in which test cells were not dying, culture medium was collected from the cultures with twice as many cells grown in test culture. Various autophagosome samples were then used for treatment of DCs.

### Mice

Female BALB/c mice aged 6–8 weeks were purchased from the National Laboratory Animal Breeding and Research Center, Taipei, Taiwan. Test mice were maintained in a standardized laminar airflow cabinet under specific pathogen-free conditions, and all manipulation and experimental protocols involving animals were approved by the IACUC office of Academia Sinica, Taipei.

### 4T1 mammary carcinoma-tumor resection model

Mice were injected subcutaneously with 4T1-Luc2 cells (5 × 10^5^ cells/50 μl PBS/mouse) into the fourth mammary fat pad under isoflurane anesthesia. Tumor growth was monitored by measuring the tumor volume, as length × (width)^2^/2. After tumors were established (180–200 mm^3^) on day 15, test mice were divided into groups (8 mice/group) and subjected to different treatments. At 16 days post tumor cell implantation, primary tumors were surgically resected and incisions closed with sutures. For immunization with DC vaccines, mice were administered with specific or control DC vaccines (1 × 10^6^ DCs/200 μl PBS/mouse) by intravenous injection at 1, 8 and 15 days post tumor resection. To monitor progression of metastatic tumors, bioluminescence signals from the 4T1-luc2 tumor cells in test mice were analyzed using a non-invasive IVIS imaging system (Calipers, Hopkinton, MA) after intraperitoneal injection of 150 mg/kg D-luciferin (NanoLight technology, Pinetop, AZ). When mice were unable to ambulate to reach food/water, or displaying signs of respiratory distress due to tumor metastasis, the mice were sacrificed (CO2 inhalation) and the date was record to calculate survival rate. The criteria used to determine humane endpoint is strictly based on Guidelines for Determining Endpoints and Humane Termination of Animals provided from the Institutional Animal Care and Use Committee (IACUC) of Academia Sinica, Taiwan (S1 File). All test mice were euthanatized by CO2 inhalation at the end of experiment.

### Western blot assay

Tumor cell lysate samples were prepared as previously described.[11, 18] 4T1 TCL protein samples were resolved by SDS PAGE using 8, 10 or 15% stepwise gels. Western blot procedures were performed as previously reported.[11] Washed blots were incubated first with specific, primary antibodies (1:1000) then with HRP-conjugated secondary antibody (1:100,000 dilution) and washed with PBST buffer. The transferred proteins were visualized with an enhanced chemiluminescence (ECL) detection assay.

### Mouse bone marrow derived dendritic cells

Mouse bone marrow derived dendritic cells (BMDCs) were generated and modified as previously described.[18] Briefly, bone marrow tissues were collected from BALB/c mice, and erythrocytes were removed. The derived bone marrow cells were cultured in 30 ml complete RPMI 1640 medium (see above) supplemented with 20 ng/mL GM-CSF and 50 μM 2-mercaptoethanol. On day 2, two-thirds of the original medium was replaced by 30 mL fresh medium. On day 5, the floating cells were gently removed and the culture replenished with fresh medium containing 20 ng/mL GM-CSF and 20 ng/mL IL-4. On day 7, the nonadherent and loosely adherent DCs in culture were harvested and used as the dendritic cell source of various vaccines. DCs were routinely generated in this manner, found mainly as immature DCs with 85% of cells expressing CD11c^+^ and displayed the typical morphologic features of DCs.[18]

### Activation of TCL- and AS-loaded DCs

Activation of BMDCs was performed as previously described.[18] Briefly, BMDCs were incubated for 2 h with various TCL samples containing 200 μg protein/ml. LPS (1 μg/ml) was then added to the medium for co-cultivation with TCL-loaded DCs for another 22 h. DCs treated with 1 μg/ml LPS only for 24 h (mDCs) were used as the vehicle control for TCL stimulation. DC samples reacted with naïve-TCLs (i.e., cell lysates collected from 4T1 tumor cells that were treated without shikonin or rapamycin stimulation) were designated as DMSO-TCL-mDCs. DC samples treated with TCLs, obtained from 4T1 cells treated for 24 h with shikonin or rapamycin for stimulation, were designated as SK-TCL-mDCs and Rapa-TCL-mDCs, respectively. Similarly, DCs reacted with autophagosome preparations Rapa-AS and SK-AS for 24 h were designated as SK-AS-mDCs and Rapa-AS-mDCs, respectively. These test DCs were then compared for their activity in stimulating T-cell proliferation by mixed lymphocyte reaction (MLR) assay for their anti-metastatic effect on 4T1 tumors in vivo.

### Mixed lymphocyte reaction assay

Mouse CD8^+^ T-cells were isolated by magnetic activated cell sorting (MACS) selection of splenocytes with CD8 microbeads (Miltenyi Biotech, Bergisch Gladbach, NRW), resulting in >98% purity and >98% viability. Co-cultivation of the DC-T cell system was performed as previously described.[11, 18] Briefly, a total of 1 × 10^5^ splenocytes or CD8^+^ T cells were co-cultured with BMDCs (at a DC/T ratio of 1:10) for 4 days in a final volume of 200 μl medium, and the BMDC-induced T-cell proliferation activity was determined by Cell Proliferation ELISA (Roche Applied Science, Indianapolis, IN).

### Flow cytometry analysis of surface and intracellular cell markers of regulatory T cells and myeloid derived suppressor cells

For detection of myeloid derived suppressor cells **(**MDSCs), peripheral blood cells from DC vaccine-immunized and control mice were collected, and cells were stained for 30 min at 4°C with antibodies against specific cell markers, including FITC-conjugated anti-mouse CD11b (for cell surface), APC-Cy7 conjugated anti-mouse Ly-6C and PE conjugated anti-mouse Ly-6G, (both for intracellular staining). All three antibodies were obtained from Biolegend, (San Diego, CA). The percentages of monocytic and granulocytic MDSCs were gated on CD11b^+^Ly-6C^+^ cells and CD11b^+^ Ly-6G^+^ cells, respectively.[19] For Treg cell detection, cells were surface stained with FITC-conjugated anti-mouse CD25 and inter-cellularly stained with APC conjugated anti-mouse Foxp3, followed by cell permeabilization with the Cytofix/Cytoperm Plus kit according to the manufacturer’s protocol for all three tested antibodies, and the reagent kit from BD pharmingen (San Diego, CA). Percentage of Treg cells was gated on CD25^+^ Foxp3^+^ cells.[20] Fluorescence signals were detected by cytometry as described above.

### Statistical analysis

Statistical analysis was performed using an unpaired, two-tailed Student’s t-test. Statistical analyses were conducted with GraphPad Prism 5.0 (San Diego, CA). Differences in tumor metastasis mouse survival rate were determined by a log-rank (Mantel-Cox) test of the Kaplan-Meier survival curves. All statistical tests were two-sided. A P-value of less than .05 was considered significant (*, *P* < .05; **, *P* < .01; ***, *P* < .001; n.s, no significance).

### Collection of data from the National Health Insurance Research Database of Taiwan

Epidemiological data for this study was collected from the Taiwan National Health Insurance Research Database (NHIRD). The database covers the health care program period from 1998 to 2010, and is managed and published by the National Health Insurance (NHI) program of Taiwan, which started in 1995 to finance health care activities and measures for all residents of Taiwan. Rapamycin was approved for clinical use on Jan. 21, 2002. The present data set from NHIRD covers all medical claims for the 22.9 million residents of Taiwan surveyed and compiled between 2002 to 2008 for cancer incidence (5 years between 2009–2013 were considered as observation period). The completeness and accuracy of the NHIRD is guaranteed (officially verifiable) by the Department of Health and the Bureau of NHI in Taiwan. Since the NHIRD consists of de-identified secondary data released to the public for research purposes, the study was exempt from a full review by the local ethics review committee at China Medical University, Taichung, Taiwan. To investigate the possible effect of rapamycin use on cancer incidence rate in human populations, data were collected and extracted from the NHIRD by Dr. T.J. Lin and Dr. W.M. Liang, a surgeon/physician and an epidemiologist, respectively. For identification and selection of Rapamycin users, all Rapamycin users recorded between year 2002 and 2008 were selected from the NHIRD database in Taiwan area, except for those patients with cancer. The selected 3,713 Rapamycin users were not detected with any cancers before 2002. Base on the distribution of age and sex for Rapamycin users, we further randomly selected the Non-rapamycin users (N = 14,852) from the same database (22.9 million residents) and also excluded those patients who were already detected to have cancer. Therefore, the cancer incidence of both selected groups, i.e., the “Rapamycin users” and “Non-rapamycin users”, were literally “0” before the year of 2002. For identification of cancer cases between year 2002 and 2008, the diagnosis of malignancies was defined by ICD-code 140–208. The study identified diagnoses of cancers with the records from the Registry of Catastrophic Illness Patient Database. To be eligible for a cancer catastrophic illness certificate, cytological or pathological reports or evidence such as additional laboratory and image studies supporting the diagnosis of cancer-including tumor marker surveys, x-ray, bone scan, computed tomography scan, or magnetic resonance imaging scan should be provided. Incidence rates of all-cause cancers were compared between the rapamycin-use subjects and then matched to the reference group using Chi-squared test and Wald test from the Poisson model.

### Ethics statement

All animal studies for this research work were performed in strict accordance with the recommendations in the Guide for the Institutional Animal Care and Use Committee (IACUC) of Academia Sinica. The protocol was approved by the IACUC of Academia Sinica *(*Protocol ID: 12-01-304) (S2 File). This research complies with the Animal Research: Reporting of *In Vivo* Experiments (ARRIVE) guidelines (S3 File).

## Results

### In vitro effect of rapamycin and shikonin on ICD and autophagy in 4T1 cells

**Fig 1:**
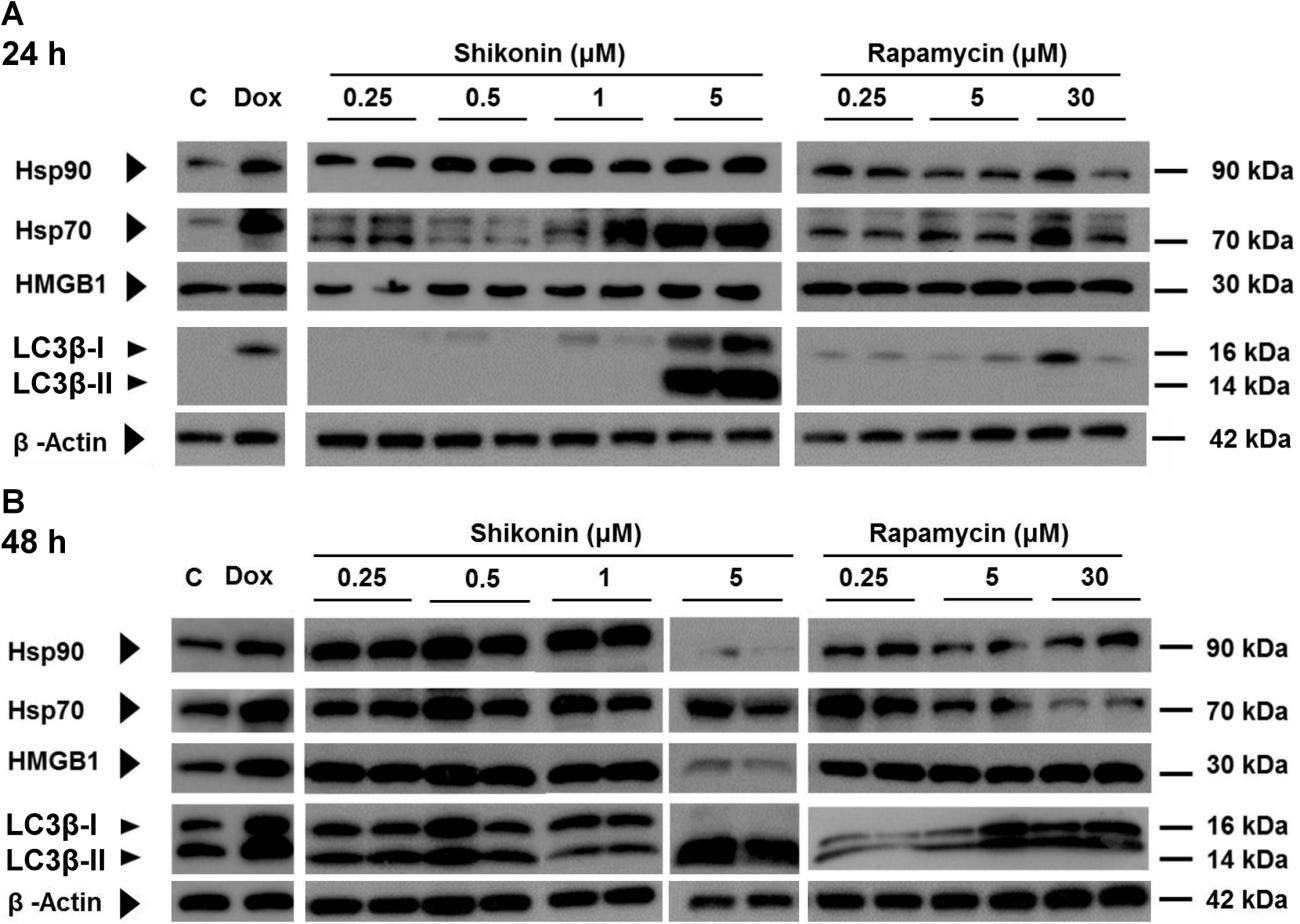
In vitro effect of shikonin and rapamycin on expression of immunogenic cell death and autophagosome markers in mouse 4T1 mammary carcinoma cells. 4T1 cells were treated with test compounds doxorubicin, shikonin and rapamycin at the indicated concentrations for (A), 24 hours or (B), 48 hours. Tumor cell lysate samples were then prepared with a standardized protocol, as described in Materials and Methods, and subjected to western blot analysis. The results are representative of three independent experiments.

Damage- or danger-associated molecular pattern molecules (DAMPs) can initiate and perpetuate immune and inflammatory response and are molecular markers of ICD. The processing of cytosolic LC3β-I to LC3β-II is a marker of autophagosome formation. For this study, cellular debris or organelle samples derived from ICD and autophagy of 4T1 mouse mammary carcinoma cells were tested for use as anti-metastasis vaccines (see below). Toward adjuvant application, we also evaluated whether the phytochemical shikonin or rapamycin could incite ICD and/or autophagic activities in 4T1 cells. Since rapamycin has been previously reported [21] to inhibit the proliferation of breast and other tumor cell lines, we were also interested in a possible direct in vitro cytotoxic effect of rapamycin on 4T1 cells. Shikonin, used as a positive control as defined by our previous study,[11] induced a high level of expression of ICD markers in 4T1 cells at 48 h post treatment (Fig 1B). However, it was effective only at the highest concentration (5 μM) when tested 24 h post treatment (Fig 1A). In contrast, little or no rapamycin effects on ICD activity were observed at 24 h post treatment. Besides, rapamycin did not induce a consistant ICD activity in a dose dependent manner, at 48 h post treatment, (Fig 1A and 1B). At 5 μM, shikonin can effectively induce LC3β-II expression (autophagosome assembly/maturation) at 24 h post treatment, whereas little or no effect of rapamycin (even at 30μM) on LC3β-II was detected (Fig 1A). Even at 48 h post-treatment with rapamycin, the expression of LC3β-II activity was only increased at high dosage (5–30 μM), as revealed by western blot assay (Fig 1B). When 4T1 cells were treated with shikonin (5 μM) for 48 h, >80% of cells were killed by the cytotoxicity of shikonin (S1 Fig). In Fig 1B, the decrease of ICD mediators suggests that the specific time period that can confer ICD activity has apparently “passed” already in the shikonin-treated 4T1 tumor cells. In contrast, high level expression of LC3β-II isoforms was still observed in 4T1 cells at 48 h post treatment with shikonin, indicating a high level of autophagosome has accumulated in the dead 4T1 cells. By taking advantage of these results, for the subsequent experiments in our study we hence induced ICD and autophagy activity in tumor cells with shikonin treatment at 5 μM for 24 h and 48 h, respectively. These results support previous reports of the anti-tumor activities against mammary carcinoma.[22, 23] In view of these results, next we conducted an *in vivo* experiment on metastasis after resection of primary 4T1 tumors in test mice.

### In vivo effect of rapamycin on metastasis of 4T1 tumors

**Fig 2:**
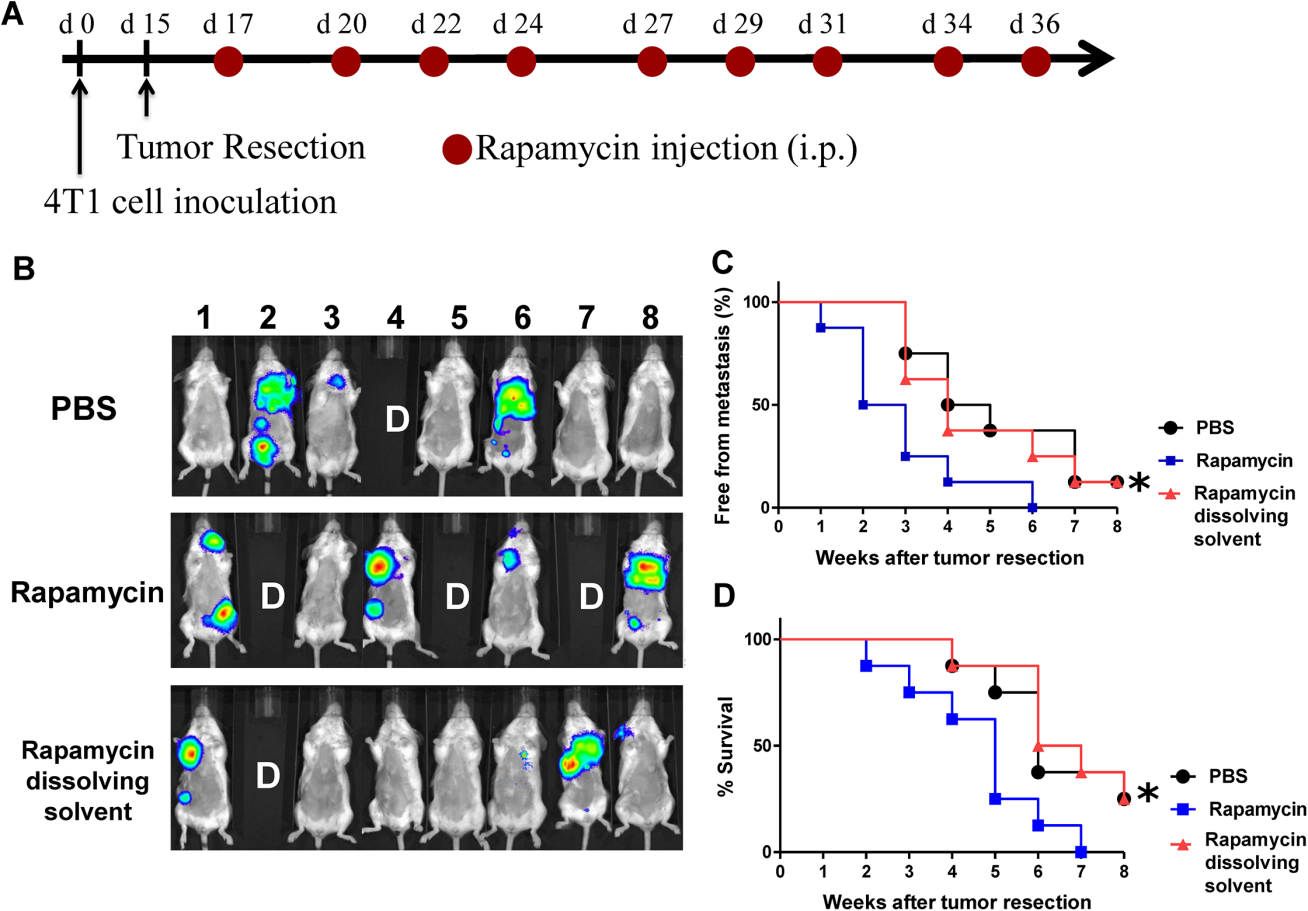
Administration of rapamycin in vivo promotes metastasis of 4T1 cells in a tumor resection mouse model. (A), Schema for treatment. Mice were injected subcutaneously with 4T1-Luc2 cells (5 × 10^5^ cells/100 μl PBS/mouse) into mammary fat pad under isoflurane anesthesia at day 0. At 16 days post tumor cell implantation, primary tumors were surgically resected. Test mice were administered with saline, Rapamycin (0.75 mg/kg) or rapamycin-dissolving solvent (vehicle control, 4% ethanol, 5% polyethylene glycol 400, and 5% Tween 80) by intravenous injection for 3 weeks (3 injections/week). (B), Representative in vivo bioluminescent images of test mice (n = 8) treated with saline, rapamycin and vehicle control solvent at 4 weeks post tumor resection. The red signals represent the highest level on the colorimetric scale. Labelling with “D” in photograph denotes the mice that had died before 4 weeks post tumor resection. (C), Quantification of tumor metastasis burden in mice treated within the indicated time course as revealed by bioluminescence imaging for luciferase activity. (D), Survival time of mice after treatment with the indicated test agents. *, the p values, P < 0.05, when the rapamycin-treated group was compared with the vehicle (dissolving solvent) control group.

To effectively evaluate the effect of rapamycin on mammary tumor metastasis, we used a bioluminescent, transgenic luciferase-labelled 4T1 mouse mammary carcinoma cell line (developed by Dr. P.W. Hsiao of Academia Sinica, Taiwan (see Materials and Methods), and the subsequent 4T1 tumor metastasis model in BALB/c mice. After resection of the orthotopic primary tumors, test mice were treated with saline, rapamycin (0.75 mg/kg) and the rapamycin-dissolving solvent (as vehicle control, containing 4% ethanol, 5% polyethylene glycol 400, and 5% Tween 80), were monitored and compared for bioluminescent images of 4T1 tumors in a time course experiment (Fig 2). In comparison, rapamycin treatment resulted in a significant increase in the level of 4T1 tumor metastasis, as compared with the saline and vehicle control treatments (Fig 2B and 2C). Consistently, rapamycin treatment also significantly decreased the life span of test mice as compared to the PBS (*P* < 0.05) and vehicle control treatment (*P* < 0.05) (Fig 2D). These results of Fig 2 together show that rapamycin treatment can effectively promote 4T1 tumor metastasis into lung, when used in combination with a resection process for primary mammary tumor. In another experiment, effect of docetaxol as a positive control for anti-4T1 metastasis in our tumor-resection study model was conducted, indicating the detectable therapeutic effect of a chemotherapy drug in our in vivo study model (S2 Fig).

**Fig 3:**
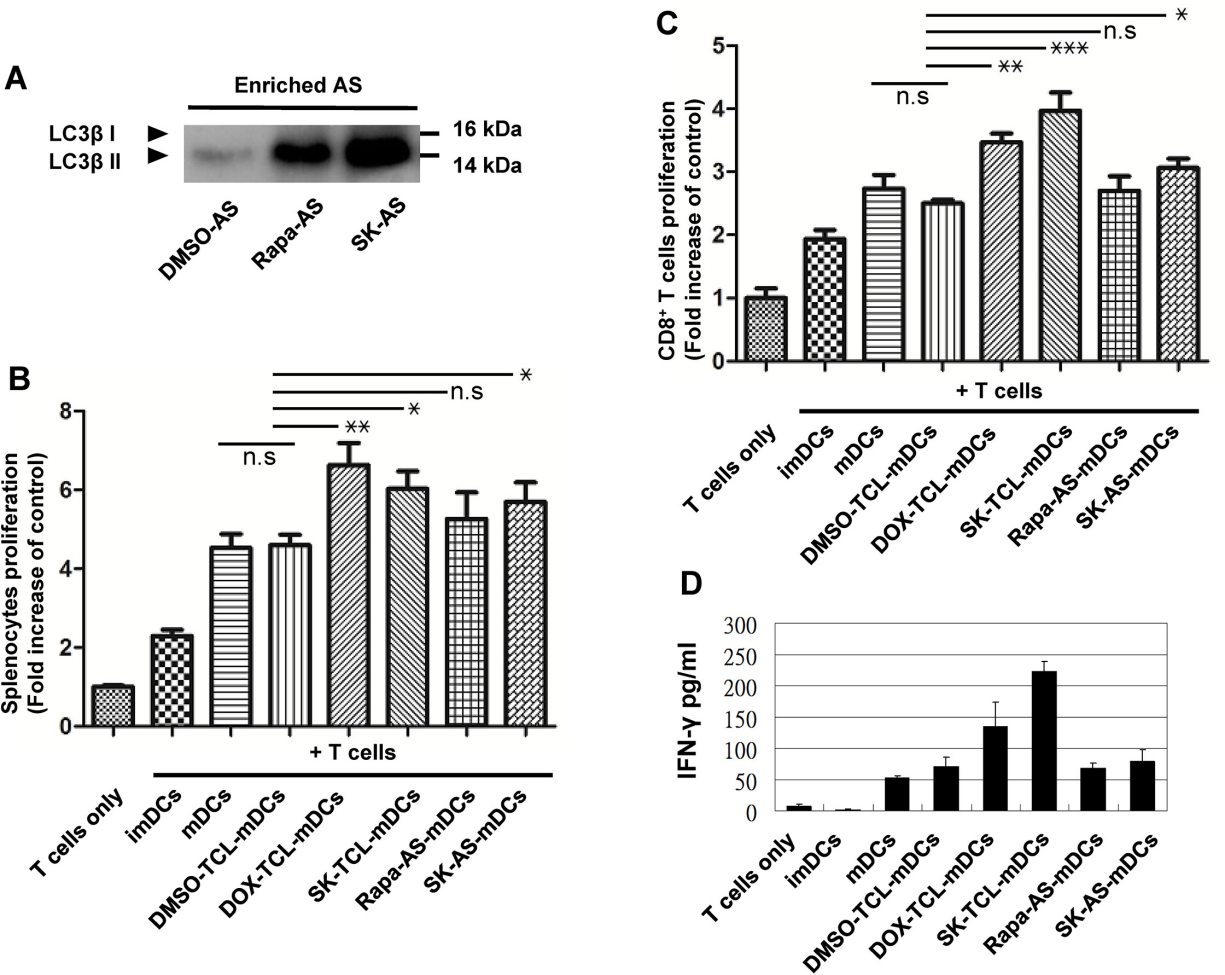
Shikonin treated tumor cell lysate- and autophagosome-activated DCs efficiently induce lymphocyte proliferation in vitro. (A), Effect of shikonin and rapamycin in treated 4T1 cells on expression of autophagosome markers. These organelle preparations were enriched, partially purified, and loaded onto DCs for vaccine preparation. Doxorubicin- and shikonin-treated 4T1 cells and the resultant-ICD derived TCLs were also used to pulse DCs for vaccine formulation. Effect of the resultant DC vaccines on in vitro proliferation of (B), Splenocytes and (C), CD8^+^ T cells, collected from syngeneic mice as responder cells, were analyzed. The p values, i.e., * *P* < 0.05; ** *P* < 0.01; *** *P* < 0.001; and n.s, no significance, were obtained between the two indicated test groups. Data represent the mean ± SE obtained from three independent experiments. (D), The secretory activity of IFN-γ in CD8^+^ T cells treated with various DC vaccine samples was evaluated be ELISA. (Rapa: Rapamycin; SK: Shikonin; AS: Autophagosome; TCL: Tumor cell lysate).

We showed previously that a much enhanced immunogenicity and the efficacy of a SK-TCL-DC cancer vaccine were due to the ICD-inducing “adjuvant” effect of shikonin in the DC vaccine formulation.[11] To evaluate the potency of induction of autophagosomes by shikonin and rapamycin and their use in formulation as a vaccine adjuvant in our present 4T1 cell system, we stimulated and collected autophagosomes and loaded them onto DCs, and the effect of the resultant DCs on T-cell proliferation was assayed. As shown in Fig 3A, rapamycin and shikonin at 5 μM resulted in very effective induction of the autophagosome marker, LC3β-Ⅱ (Rapa-AS and SK-AS), in 4T1 cells, as compared to a control. Autophagosome and TCL preparations were hence isolated from 4T1 cells treated or not treated with rapamycin, doxorubicin or shikonin, and further tested for stimulation of splenocyte and CD8^+^ T cell proliferations. Doxorubicin-TCL loaded mature DCs exhibited the highest levels of stimulation of splenocyte proliferation; this was followed by SK-TCL-loaded mature DCs, shikonin-induced autophagosome (SK-AS) loaded mature DCs (SK-AS-mDCs), then rapamycin-induced autophagosome loaded mDCs (Rapa-AS, mDCs) (Fig 3B). Stimulation of CD8^+^ T cell proliferation among the treatment groups was in the following order, from highest level of proliferation to lowest level: SK-TCL-loaded mature DCs, shikonin- and then rapamycin—induced autophagosome (AS)-loaded mature DCs (Fig 3C). In addition, the secretory activity of IFN-γ in CD8^+^ T cells treated with various DC vaccine samples was evaluated by ELISA. Consistently, T cells pulsed with SK-TCL-mDCs expressed higher levels of IFN-γ (Fig 3D), and this was followed by stimulation with Dox-TCL-DCs. However, both SK-AS-mDCs and Rapa-AS-mDCs did not confer a significant effect on this activity of test CD8^+^ T cells. Our data hence demonstrate that the SK is an active phytochemical on induction of ICD activity in treated tumor cells and responsible for the subsequent tumor immunogenicity of cytotoxic T cells. These results were representative of three independent experiments and we, therefore, propose with confidence that in our system, rapamycin, unlike shikonin and doxorubicin, has very limited capability to augment the efficacy of our tumor cell-based cancer vaccines.

### DC vaccines pulsed with shikonin-treated TCL (SK-TCL-DC) significantly suppressed rapamycin-enhanced metastasis of mammary tumor cells

**Fig 4:**
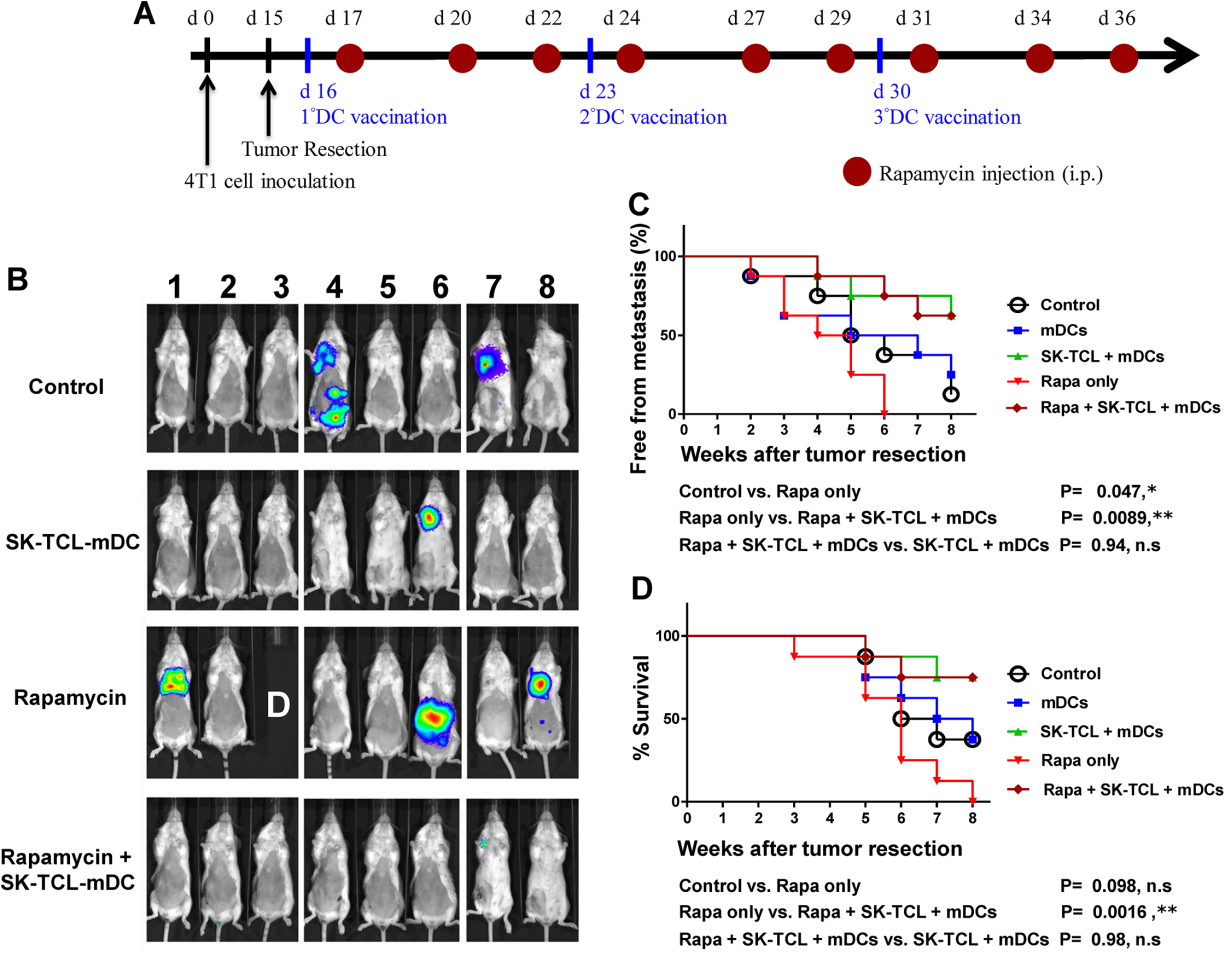
Vaccination with SK-TCL-activated DCs effectively suppresses rapamycin-promoted metastasis of 4T1 cells after resection of the primary tumor. (A), Mice were injected subcutaneously with 4T1-Luc2 cells (5 × 10^5^ cells/100 μl PBS/mouse) into the mammary fat pad at day 0. At 16 days post tumor cell implantation, orthotopic primary tumors were surgically resected. Test mice were administered with control (saline) or rapamycin by intraperitoneal injection, and subsequently with or without vaccination by SK-TCL-mDCs by intravenous injection at indicated time points. (B), Representative bioluminescent images of tumor-resected mice (n = 8) in each group shown at 4 weeks post tumor resection. The red signal represents the highest level on the colorimetric scale. Labelling with “D” in photograph denotes the mice that had died before 4 weeks post tumor resection. (C), Quantification of tumor metastasis by measuring luciferase activity in p/s/cm^2^/sr in mice revealed along the indicated time points (n = 8). (D), Survival of test mice after different treatments. Labelling symbol for the control mouse group was intentionally marked with relatively large open circles in order to allow a clear indication of data sets obtained from each experimental group. *, values of *P* < 0.05; **, *P* < 0.01, were obtained between the Rapa only and Rapa + SK-TCL + mDCs groups. Similar results were obtained from three independent experiments.

The results of Fig 3 further support the findings of our previous study using B16 cells [11] by once again demonstrating that shikonin-treated tumor cell lysate can be effectively employed to pulse and enhance mature dendritic cells (SK-TCL-mDC, Fig 3B and 3C) for therapeutic vaccination against metastasis of 4T1 tumors. As seen in Fig 2, we showed that *in vivo* treatment of mice with rapamycin promotes 4T1 tumor metastasis in our test mouse system. Therefore, next we were interested in whether rapamycin-promoted 4T1 cell metastasis after resection of the original primary tumor, could be suppressed by treatment with the SK-TCL-mDC cancer vaccine (Fig 4). In this experiment, an “anti-metastasis protocol” was designed in which the test DC vaccine was delivered on the very next day of tumor resection (Fig 4A). Bioluminescent imaging analysis of mice treated with control (saline) and different vaccine adjuvant formulations (mDCs only, SK-TCL-mDC, rapamycin only, and rapamycin + SK-TCL-mDC) were scored and analyzed. Consistent with the result of Fig 2, rapamycin treatment significantly increase the level of 4T1 tumor metastasis, as compared with the vehicle control treatment (*P* < 0.05) (Fig 4C), In contrast, SK-TCL-mDC treatment drastically reduced the tumor incidence and the rapamycin-promoted tumor metastasis, as evidenced by the lowest activity for metastasized tumors (Fig 4B and 4C). Tumor metastasis was suppressed by SK-TCL-mDC vaccination to a level of 62.5% at 2 months post tumor resection, as compared with the control group (*P* < 0.01) (Fig 4C). The level of anti-metastatic effect for rapamycin + SK-TCL-mDC treatment was similar to, i.e., not statistically different from, that of SK-TCL-mDC treatment (*P* = 0.94, n.s) (Fig 4C). The mean survival period of test mice with lung metastasis was 5, 12, 4 and 10 weeks for the control, SK-TCL-mDC, rapamycin, and rapamycin + SK-TCL-mDC group, respectively (Fig 4D). Co-treatment of rapamycin and SK-TCL-mDC vaccination (Rapa + SK-TCL-mDCs) significantly increased the life span of mice as compared to the vaccine-untreated (i.e., rapamycin alone) mice (*P* < 0.001), or the control group mice (i.e., untreated with DC vaccine and untreated with rapamycin) (Fig 4D). Together, these results clearly demonstrate that SK-TCL-mDC can effectively inhibit the initiation and progression of highly metastatic 4T1 malignancies promoted by *in vivo* administration of rapamycin in test mice.

### Epidemiological and statistical analyses of cancer incidence in rapamycin user and non-user human populations

As we found that rapamycin promoted 4T1 tumor growth *in vivo* and induced autophagy in treated tumor cells *in vitro*, we considered it important to investigate whether rapamycin-stimulated tumor incidence may also be relevant in humans. The National Health Insurance (NHI) program of Taiwan has been implemented for 15 years, and a highly comprehensive and exhaustive NHI database is accessible for public research and documentation. As seen in Table 1 and the detailed information provided in its footnote, by scoring a population of 3,713 rapamycin users (all enrolled between 2002 and 2008) and 14,852 randomly selected, non-rapamycin users over the same time period, with time-frames defined as “incubation-time” for disease occurrence and other relevant definitions, and by using stringent classifications and restrictions on data analyses, we observed that during a seven year period of rapamycin use, the cancer incidence rate (for all types) of rapamycin users and non-users was 5.14% (191/3713) and 1.84% (274/14852), respectively (Table 1). This approximately 2.79 fold difference in cancer incidence rate, we consider, is highly statistically significant, as determined using a Chi-square test (*p-value* < 0.0001). This difference may also have health care and societal applications, in terms of population size, public health concerns and chemo-prevention measures. Based on this analysis, we suggest that there is a correlation between rapamycin usage (mostly among kidney transplantation patients) and an increased cancer incidence rate in humans, at least in the general population of Taiwan.

**Table 1:** Demographics and cancer incidence rates for rapamycin-treated vs.-untreated human subjects between year 2002–2008.

	Test Group (Rapamycin users) N = 3,713	General Population (Non-rapamycin users) N = 14,852
**Age, n (%)**		
< 20 years old	162(4.36)	637(4.29)
20–30 years old	407(10.96)	1,618(10.89)
30–40 years old	738(19.88)	2,987(20.11)
40–50 years old	1,184(31.89)	4,710(31.71)
50–60 years old	937(25.24)	3,752(25.26)
> 60 years old	285(7.68)	1,148(7.73)
**Sex, n (%)**		
Male	1,990(53.60)	7,960(53.60)
Female	1,723(46.40)	6,892(46.40)
Mean period of follow-up (in days)	1750.72±580.14	1865.16±506.42
Days of Rapamycin use per person-year	126.25	-
Cancer, n (%)		
No	3,522(94.86)	14,578(98.16)
Yes	**191(5.14)**	**274(1.84)**
Cancer incidence per 100,000 person-years ^a^	**1,073.00**	**361.28**

To investigate possible effect of rapamycin use on cancer incidence rate in human populations, data were collected and extracted from the National Health Insurance Research Database (NHIRD) of Taiwan. The information on 3,713 individuals who were subjected to rapamycin treatment (medical code: B023057151, B023363100) between 2002–2008 were obtained through NHI database from the records of outpatients, inpatients and pharmacies, all of which were enrolled and connected with the National Health Insurance system of Taiwan. The index day was assigned on the first day when rapamycin was used. Controls were selected from the ID file enrolled in the NHI program, which were matched with rapamycin users at a ratio of 1:4 by age, gender and index day. The diagnosis of malignancies was defined with the ICD-9-CM codes of 140–208. All enrolled subjects with a history of malignancies before the index day were excluded. These two cohorts were followed up until the development of cancer, termination of NHI program, or the end of the period of observation at December 31, 2010 to ensure a minimum follow up period of two years for every enrolled subject.

^a^ Cancer incidence is expressed as the number of cancers per 100,000 person-years.

### Involvement of MDSCs and Tregs in rapamycin and DC vaccine effects

**Fig 5:**
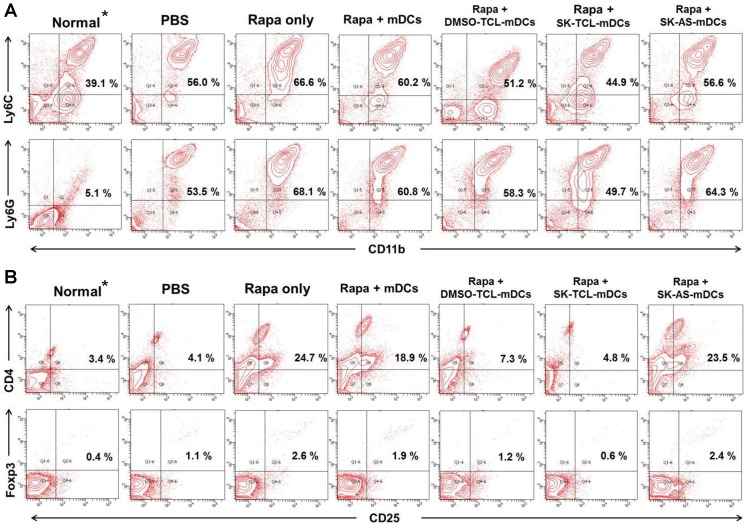
SK-TCL-activated DCs can suppress rapamycin-enhanced MDSC and Treg cell expansion in vivo. (A), Effect of various DC vaccines on population change of monocytic and granulocytic MDSCs in blood of test mice, quantified using flow cytometry. The monocytic and granulocytic subpopulations of MDSCs were analyzed using a FACS system and DIVA software as gated on CD11b^++^Ly6C^++^ and CD11b^++^Ly6G^++^ cells, respectively. (B), Suppression of Treg cells in rapamycin-treated mice which were subsequently immunized with or without test DC vaccines. Six days post the last vaccination, test cells were isolated from the blood of normal and various rapamycin-treated mice. The populations of Treg cells were quantified using FACS DIVA analysis and gated on CD4^+^CD25^+^ (pan-phenotype of Treg cells) and Foxp3^+^CD25^+^ (phenotype of a specific Treg population with immunosuppressive functions) cells. Normal* denotes mice that were not inoculated with 4T1 tumor cells and hence were not tumor-bearing mice.

The importance of Tregs and myeloid derived suppressor cells (MDSCs) in the regulation of tumor growth is well documented.[24–27] We, therefore, went on to analyze the possible effect of the test vaccines on inhibition of MDSC and Treg cells in immunized animals. There are two major subsets of MDSCs, monocytic (CD11b^+^Ly6C^+^) and granulocytic (CD11b^+^Ly6G^+^) MDSCs. The monocytic cells were previously shown to inhibit T-cell proliferation in vitro, whereas granulocytic MDSCs were reported not to inhibit such activity.[27] In our present study the populations of monocytic and granulocytic MDSCs in blood samples of various DC-vaccinated mice were analyzed and compared at 3 weeks post tumor resection. As shown in Fig 5A, compared with the PBS (vehicle)-treated mice, the CD11b^+^Ly6C^+^ and CD11b^+^Ly6G^+^ MDSC populations in rapamycin-treated mice were significantly increased, from 56.0% to 66.6% and from 53.6% to 68.1%, respectively. In contrast, DC vaccination (SK-TCL-mDCs) in combination with the *in vivo* administration of rapamycin subsequently reduced CD11b^+^Ly6C^+^ and CD11b^+^Ly6G^+^ MDSC populations to 44.9% and 49.7% (33% and 28% decrease), respectively. These levels were even lower than those (56.0% and 53.5% for m-MDSC and g-MDSC, respectively) detected in test mice treated with PBS only, without DC vaccine treatment. However it is important and interesting to note here that the drastic expansion of the granulocytic MDSCs (CD11b^+^Ly6G^+^) after 4T1 tumor inoculation (from 5.1% in normal mice to 53.5% in PBS treated mice) was not able to be reversed by the combined treatment of rapamycin and SK-TCL-mDCs (i.e., 49.7%), suggesting that a portion of the tumor metastasis-mediated granulocytic MDSC expansion can not be rescued by the activity of test DC vaccine. This specific portion or level of g-MDSC expansion is apparently not associated or caused by the same mechanism(s) related to the interference of immune cell activities by rapamycin. And, we hence term this activity as rapamycin-independent g-MDSC expression activity during tumor metastasis. This class of tumor-promoted g-MDSC activity is apparently refractile to our present TCL-DC based vaccine therapy. Future studies are needed to dissect the mechanistic mode of action for this class of MDSC activity. When rapamycin treatment was combined with three other DC-vaccine groups (i.e., mDCs, DMSO-TCL-mDCs and SK-AS-mDCs), much less suppressive effect was detected for the rapamycin-enhanced populations of monocytic and granulocytic MDSC cells, as compared to the combined treatment of rapamycin and SK-TCL-mDCs (Fig 5A). These results suggest that only the SK-TCL-mDC vaccination was able to substantially reverse the rapamycin-enhanced monocytic as well as granulocytic MDSC populations.

Treg cells, a subpopulation of the CD4^+^ T cells expressing CD25 and transcription factor FOXP3, are known to play a key role in promoting growth and progression of tumors by inhibiting specific immune responses against the host tumors.[28, 29] As shown in (Fig 5B), as compared with the PBS (control)-treated group, the CD4^+^CD25^+^ and CD25^+^Foxp3^+^ Treg cell populations in the blood of the rapamycin-treated (Rapa only) mice were upregulated, from 4.1% to 24.7%, and from 1.1% to 2.6%, respectively, and these levels were strongly suppressed in mice co-treated with the SK-TCL-mDC vaccine (4.8% and 0.6%, respectively). Furthermore, these levels of both CD4^+^CD25^+^ and CD25^+^Foxp3^+^ cells in (rapamycin + SK-TCL-mDC)-treated mice were quite comparable to the levels detected in normal (i.e., not tumor-bearing) mice (i.e., 3.4% and 0.4%, respectively). Our results therefore strongly suggest that a phytochemical shikonin-activated DC vaccine formulation, SK-TCL-mDC, can exhibit a significant beneficial effect on suppression of rapamycin-enhanced Treg cell and MDSC expansion under in vivo conditions.

## Discussion

In this study, we selected an orthotopic 4T1 primary tumor growth and a subsequent tumor-resection mouse model to conduct an investigation into mammary tumor metastasis. Using this clinically-relevant model we first demonstrated that, as expected, the orthotopic 4T1 primary tumors and their metastases are highly sensitive to docetaxol, a chemotherapy drug (S1 Fig). To our surprise, whereas docetaxol very effectively suppressed the metastasis of 4T1 cells in test mice, rapamycin treatment greatly promoted the metastatic activity (Fig 2C). Consistent with the tumor-imaging data (Fig 2B), the survival rate (animals free from metastasis) and survival time of mice with lung metastasis as a result of rapamycin were also drastically reduced (Fig 2D). We consider that the striking, opposite effects of decetaxol (a common chemotherapy drug for human breast cancers) and rapamycin (an organ transplantation drug and a candidate chemotherapy drug) [30] observed in the present tumor model, is a highly significant and striking finding that may have significant future implications for human cancer treatments.

A number of studies on rapamycin, testing its effect in aging mice, showed they are prone to develop multiple spontaneous tumors and metastatic-like nodules in the lungs.[31] In these studies rapamycin was fed orally, while in our present study, the rapamycin was administered intraperitoneally (ip). Rapamycin has been shown to extend the life span through delay of cancer in some previous studies,[32] whereas in our present study test mice died earlier via tumor metastasis. This apparent contradiction may due to the fact that rapamycin administered intraperitoneally may confer a drastically different effect on the metastases than rapamycin administered orally, presumably because rapamycin might be metabolized differently, resulting in different metabolic products of the drug. Interestingly, a recent study [33] showed a quite similar result as seen in our present study, instead of Shikonin-treated tumor cell lysates, they employed sunitinib to offset the metastasis-promotive effect of rapamycin. Importantly, in their study rapamycin was also given via ip injection. Therefore future study is needed to distinguish the possible differential in vivo effect of rapamycin when delivered orally vs. intraperitoneally.

Because of the critical role of the mTOR signaling pathway in tumor cell growth and relevant anti-cancer activities, specific rapamycin analogues or derivatives have been evaluated by the FDA for treating renal cell carcinoma.[2] A phase III clinical trial study was also conducted on breast cancer patients, in which sirolimus was tested in combination with letrozole, an aromatase inhibitor. However, this combination was not found to confer a more beneficial effect than the use of letrozole alone.[8] These clinical studies on renal and breast carcinomas, although not showing efficacy, also did not reveal or report any potential “side effects”. Our findings demonstrating that rapamycin promoted mammary tumor metastasis in mice (Fig 2) seem to be in direct contradiction to the anticancer effects of rapamycin derivatives that were predicted for these two clinical trial studies. [2, 8]

We consider that the promotion of metastasis observed in the current study may be due to the specific effect of rapamycin on modulation of specific T cell types, e.g., Th1,[15] Treg cell and MDSC populations.[34] Our data indicate that our DC-based immune therapy was indeed able to confer a strong inhibition of Treg cell and MDSC expansion (Fig 5). We, therefore, suggest that, toward the various tumor- microenvironments, a rapamycin/DC vaccine combined treatment may translate into an activity-neutralizing, immune cell type-specific therapeutics in clinical cancer therapy. In addition to our laboratory experimental data, we conducted an epidemiological study on the potential risks of rapamycin, usage in human clinical situations. Our results from analysis of the data from the NHIRD database (Table 1) show that the human cancer incidence rate in rapamycin-users is significantly higher (≧2.79 folds) than that of non-rapamycin users, agreeing with the results from the mouse study. Whether or not this rate increase was negatively related to the “survival time” of test patients is not known, since the data on personal details and follow-ups of these rapamycin-user cancer patients and their counterpart population as non-user cancer patients are not available to us from the current data base of our study. Future clinical/epidemiology studies are therefore needed for such follow-up studies. Our present study in the mouse model revealed a correlation between the negative effect of rapamycin and a counteractive positive effect of a dendritic cell-based vaccine in cancer therapy experiment. This effect, detected in a mouse mammary tumor resection and subsequent metastasis experiment, may be considered as partially supported by a human cancer epidemiology study shown in Table 1. It is important for us to recognize here that we are not certain, at the present time, whether such a correlation is due to a direct opposite effects on specific immune suppressor cells, i.e., as a “direct mechanistic involvement” in pro- versus anti- metastatic activity, or an “indirect or parallel association” of these two effects. Therefore future study is needed to distinguish these two possibilities.

Western bolting with an anti-HSP70 antibody, which can react with two protein bands at 82 and 62 kDa, indicates the presence of two members of the heat shock protein family HSP70. [35] The functions of HSP70 proteins as chaperones aiming at the stabilization of cellular physiology under environmentally stressed conditions are well characterized. HSP70s are generally believed to be functionally overlapping, the main difference between different isoforms is apparently in their spatio-temporal expression, especially in response to stress conditions.[36] In our present study, after 4T1 cells were treated with shikonin or rapamycin at the indicated concentrations for 24 hours, the expression of two HSP70 isoforms were found to be transformed into a single form after the concentration of shikonin was increased to 5 μM. This transition, we speculate, may reflect a cellular stress associated with the onset of autophagosome and/or immunogenic tumor cell death activities. As for the transition of a single form HSP70 into two isoforms, we consider, it may also reflect a sub-cellular stress resulting from treatment with rapamycin at an increased concentration. Future experiment is needed to verity these possibilities.

In previous studies, rapamycin treatment of cells in culture has been broadly used for induction of cell autophagy.[37] In addition to the induction of DAMP proteins and immunogenic cell death activity, the phytochemical shikonin can also induce autophagy in 4T1 mammary carcinoma cells (Fig 3A). We showed that autophagosome and ICD preparations can be effectively prepared, collected and isolated as organelles from both rapamycin- and shikonin-treated 4T1 cells in culture. For use as adjuvant in DC-based tumor vaccines, the DOX-TCL- and SK-TCL-loaded mature DCs exhibited the highest activity in promoting splenocyte and CD8^+^ T-cell proliferations, the rapamycin-derived autophagosome loaded mDCs (Rapa-AS-mDCs, Fig 3) exhibited the least effect. Therefore, although rapamycin treatment of 4T1 tumor cells can result in high expression of autophagosomal LC3β-II (Fig 3A), the rapamycin-resultant autophagosome preparation had little effect in vivo on activating DC-mediated DC8^+^ T cell and splenocyte proliferations (Fig 3B and 3C). Together, these results lead us to conclude that, shikonin, but not rapamycin may have the potential for further development as an adjuvant component for DC-based cancer vaccines. This finding on rapamycin contrasts with that reported by Garg et al.,[38] suggesting future, direct, side-by-side comparison may be necessary.

In our previous study, we showed that a SK-TCL-loaded DC vaccine can activate antigen-specific Th1 cells and subsequently augment cytotoxic T lymphocyte (CTL) activity, resulting in the growth retardation of B16 melanoma and prolonging mouse survival time.[11] In this study, Fig 4B clearly showed that SK-TCL-mDC markedly inhibited the incidence rate and progression of rapamycin-promoted 4T1 tumor metastasis. Rapamycin clearly activated the expansion or/and differentiation of Treg cells and MDSCs (Fig 5). This result suggests that the in vivo effect of rapamycin can be counter-balanced mechanistically, via regulation of specific immune cell types, by a SK-TCL-mDC vaccine-mediated immunity. With future support from experimental/clinical studies of the human systems (see below), we consider our current ex-vivo DC vaccine approach may provide a useful strategy for a combinational use with rapamycin in clinics, to reverse the promotion of tumor metastasis by rapamycin. With such possibility, it is important to note that 4T1 mouse tumor model has been previously reported for its expansion of MDSC in tumorigenesis. At the late stage of tumor growth, 80–90% of leukocyte cells in the blood of 4T1 tumor bearing mice were found to be MDSCs.[39] For the case of human cancer patients, this percentage of MDSCs has been reported to be considerably lower, about 22% in stage Ⅳ cancer patient.[40] As shown and known for many cellular immune and tumor immune systems, substantial differences in various leukocyte, cytokine/chemokine populations/activities can be observed between the human versus the mouse systems. Therefore for potential clinical application, our result on this MDSC expansion in tumor bearing mice vs. human cancer patients needs to be carefully addressed in future investigations.

**Fig 6:**
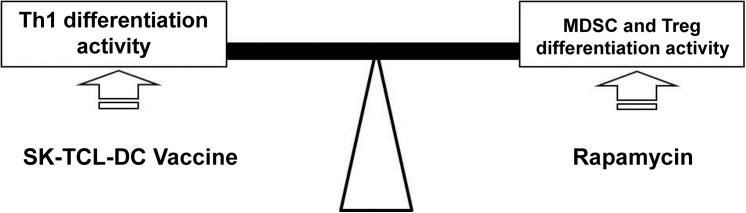
Hypothetical model depicting a possible interaction between in-vivo rapamycin treatment and ex-vivo DC-based immunotherapy for effective balance/maintenance of different T lymphocyte activities in suppression of mammary carcinoma metastasis.

Relevant to these rapamycin-mediated Treg cell activities,[41] in our previous and present studies we showed that shikonin can effectively induce the expression of specific DAMPs, which can in turn activate caspase cascades in treated B16 and 4T1 cells.[11] In combination with DAMPs, shikonin-induced TCL can activate DCs to a full level of phenotypic and functional maturation, which in turn can promote the development of Th1 cells and the induction of cytotoxic T lymphocyte activities and their secretory expression of a specific Th1 cytokine, IFN-γ (Fig 3D). Together, these activities, and in combination with the decrease in MDSC and Treg cell activities (Fig 5), we believe, may contribute to a highly efficacious retardation of tumor growth and prolong the survival of test mice. Based on these findings, we propose a hypothetical model (Fig 6) to describe the possible immuno-cellular mechanisms by which a dendritic cell-based immunotherapeutic vaccine, when administered concomitantly with rapamycin in vivo, may provide a beneficial effect to counter balance, offset and maintain the activities of specific subsets of T lymphocytes, useful for treatment of cancer patients. Among the different phytochemicals tested in this study and our previous investigation,[11] shikonin shows the highest activity in promotion of ICD in treated tumor cells, we therefore consider that further clinical evaluation of shikonin in human immune cell-based therapy is warranted.

## Supporting Information

S1 Fig Cytotoxic effect of shikonin on 4T1 tumor cells. 4T1 cells were dispensed in 96-well plates (1×10^4^ cells/well) and incubated with various concentrations of SK (0–10 μM) for 24 h (A) or 48 h (B). Percentages of cell viability, determined by MTT assay, were normalized to vehicle control group (0.1% DMSO). All treatments were performed in triplicate cultures. Data are representative of three independent experiments.
(TIFF)

S2 Fig Administration of rapamycin in vivo promotes metastasis of 4T1 cells in a tumor resection mouse model. Mice were injected subcutaneously with 4T1-Luc2 cells (5 × 10^5^ cells/100 μl PBS/mouse) into mammary fat pad under isoflurane anesthesia at day 0. At 16 days post tumor cell implantation, primary tumors were surgically resected. Test mice were administered with saline, Rapamycin (0.75 mg/kg) or Docetaxol (10mg/kg) by intravenous injection for 3 weeks (3 injections/week). (A), Representative in vivo bioluminescent images of test mice (n = 6) at 4 weeks post tumor resection. The red signals represent the highest level on the colorimetric scale. D, mice was died before 4 weeks post tumor resection. (B), Quantification of tumor metastasis burden in mice treated within the indicated time course as revealed by bioluminescence imaging for luciferase activity. (C), Survival time of mice after treatment with the indicated test agents. The p values, P < 0.05, when the rapamycin-treated group was compared with the vehicle control group.
(TIFF)

S1 File Guidelines for determining endpoints and humane termination of animals. (PDF)

S2 File Approval letter. This is to certify that the animal protocol by the following applicant has been evaluated and approved by the Institutional Animal Care and Use Committee of Academia Sinica (AS IACUC).
(PDF)

S3 File ARRIVE checklist. (PDF)
