# Supplementary figures and images for: Rapamycin Promotes Mouse 4T1 Tumor Metastasis that Can Be Reversed by a Dendritic Cell-Based Vaccine
Source: PLoS One. 2015 Oct 1;10(10):e0138335. doi: 10.1371/journal.pone.0138335 (PMC4591294; doi:10.1371/journal.pone.0138335)

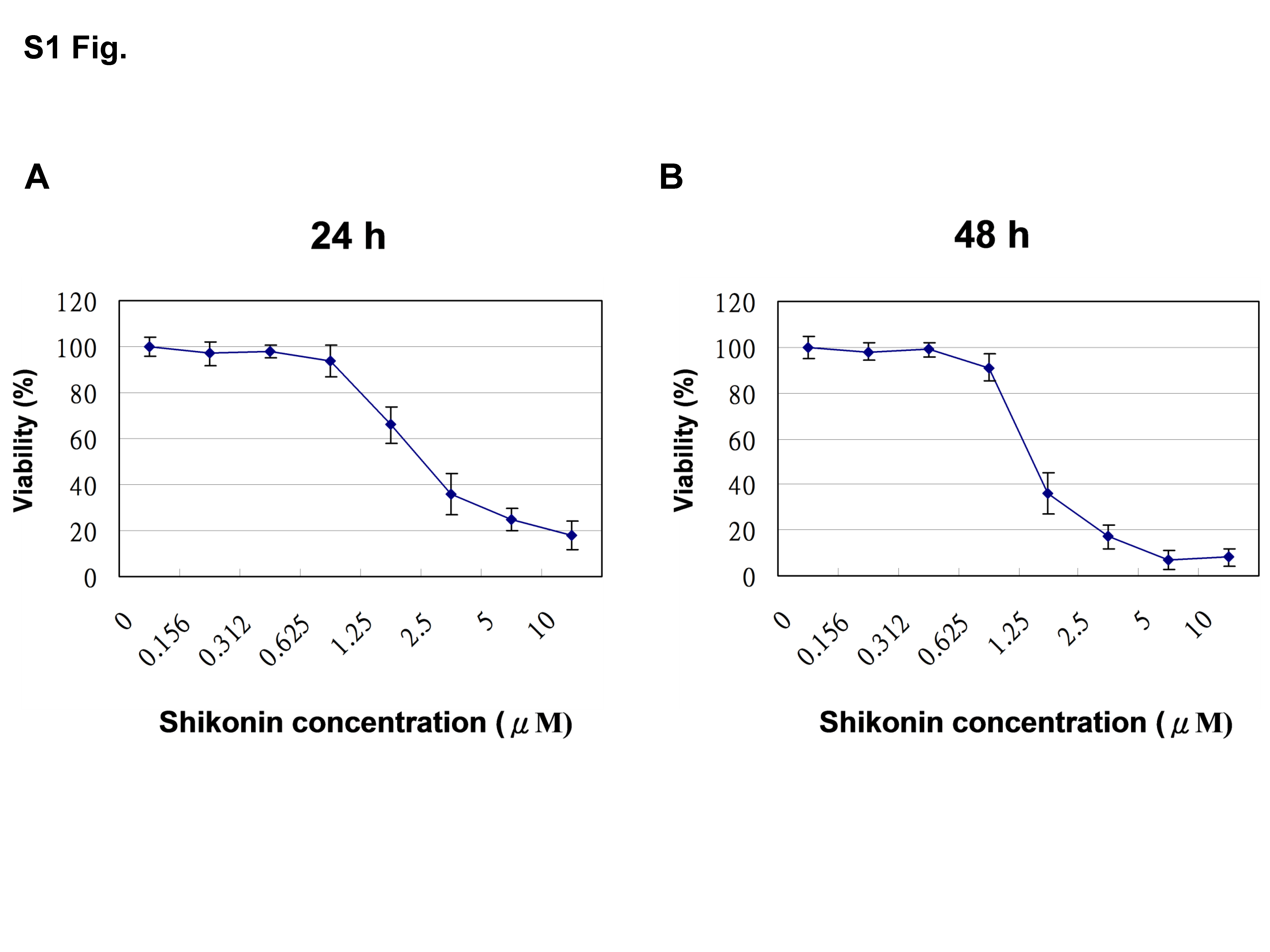

Supplement: S1 Fig — 4T1 cells were dispensed in 96-well plates (1×10^4^ cells/well) and incubated with various concentrations of SK (0–10 μM) for 24 h (A) or 48 h (B). Percentages of cell viability, determined by MTT assay, were normalized to vehicle control group (0.1% DMSO). All treatments were performed in triplicate cultures. Data are representative of three independent experiments. (TIFF) [file pone.0138335.s001.tiff]

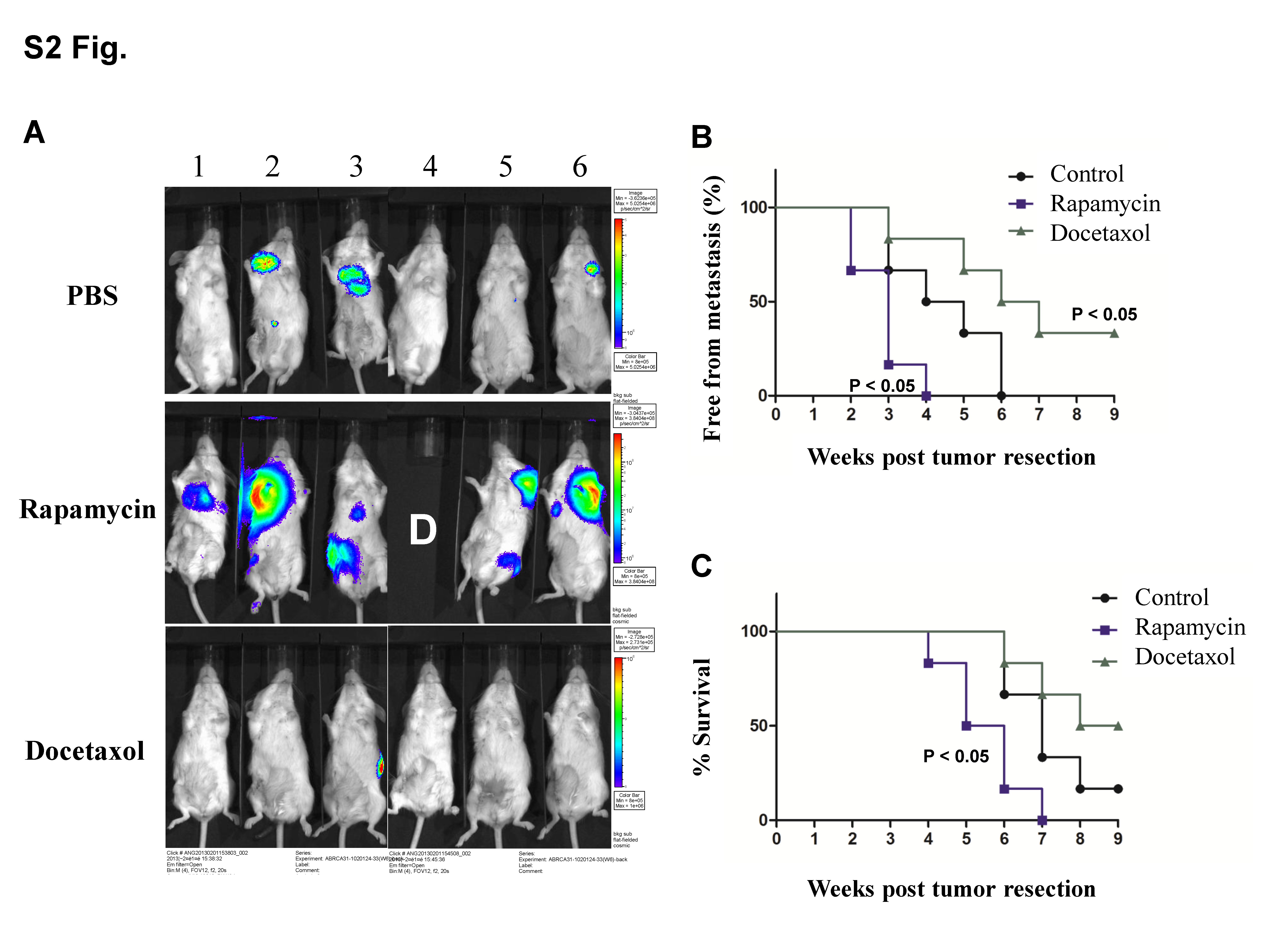

Supplement: S2 Fig — Mice were injected subcutaneously with 4T1-Luc2 cells (5 × 10^5^ cells/100 μl PBS/mouse) into mammary fat pad under isoflurane anesthesia at day 0. At 16 days post tumor cell implantation, primary tumors were surgically resected. Test mice were administered with saline, Rapamycin (0.75 mg/kg) or Docetaxol (10mg/kg) by intravenous injection for 3 weeks (3 injections/week). (A), Representative in vivo bioluminescent images of test mice (n = 6) at 4 weeks post tumor resection. The red signals represent the highest level on the colorimetric scale. D, mice was died before 4 weeks post tumor resection. (B), Quantification of tumor metastasis burden in mice treated within the indicated time course as revealed by bioluminescence imaging for luciferase activity. (C), Survival time of mice after treatment with the indicated test agents. The p values, P < 0.05, when the rapamycin-treated group was compared with the vehicle control group. (TIFF) [file pone.0138335.s002.tiff]
